# Construction of a Novel Shuttle Vector for *Tetragenococcus* species based on a Cryptic Plasmid from *Tetragenococcus halophilus*

**DOI:** 10.4014/jmb.2209.09024

**Published:** 2022-12-26

**Authors:** Min Jae Kim, Tae Jin Kim, Yun Ji Kang, Ji Yeon Yoo, Jeong Hwan Kim

**Affiliations:** 1 Division of Applied Life Science (BK21 Four), Graduate School, Gyeongsang National University, Jinju 52828, Republic of Korea; 2 Institute of Agriculture and Life Science, Gyeongsang National University, Jinju 52828, Republic of Korea

**Keywords:** *Tetragenococcus halophilus*, pMJ32E, shuttle vector, RCR plasmid

## Abstract

A cryptic plasmid (pTH32) was characterized from *Tetragenococcus halophilus* 32, an isolate from jeotgal, Korean traditional fermented seafood. pTH32 is 3,198 bp in size with G+C content of 35.84%, and contains 4 open reading frames (ORFs). *orf1* and *orf2* are 456 bp and 273 bp in size, respectively, and their translation products showed 65.16% and 69.35% similarities with RepB family plasmid replication initiators, respectively, suggesting the rolling-circle replication (RCR) mode of pTH32. *orf3* and *orf4* encodes putative hypothetical protein of 186 and 76 amino acids, respectively. A novel *Tetragenococcus*-*Escherichia coli* shuttle vector, pMJ32E (7.3 kb, Em^r^), was constructed by ligation of pTH32 with pBluescript II KS(+) and an erythromycin resistance gene (ErmC). pMJ32E successfully replicated in *Enterococcus faecalis* 29212 and *T. halophilus* 31 but not in other LAB species. A *pepA* gene, encoding aminopeptidase A (PepA) from *T. halophilus* CY54, was successfully expressed in *T. halophilus* 31 using pMJ32E. The transformant (TF) showed higher PepA activity (49.8 U/mg protein) than *T. halophilus* 31 cell (control). When *T. halophilus* 31 TF was subculturd in MRS broth without antibiotic at 48 h intervals, 53.8% of cells retained pMJ32E after 96 h, and only 2.4% of cells retained pMJ32E after 14 days, supporting the RCR mode of pTH32. pMJ32E could be useful for the genetic engineering of *Tetragenococcus* and *Enterococcus* species.

## Introduction

*Tetragenococcus* species (sp.) are halophilic lactic acid bacteria (LAB) showing the optimum growth in culture medium with 5-10% NaCl, and some species can grow up to 20% NaCl [1]. *Tetragenococcus* species have been isolated from fermented foods with high salinities such as doenjang, fish sauce, jeotgal, and soy sauce [2-5]. *T. halophilus*, the most well-known species, was previously known as *Pediococcus halophilus*, and reclassified as *T. halophilus* based on 16S rRNA sequence comparisons with other closely related LAB [6]. To date, 5 species are known and they are *T. halophilus*, *T. muriaticus*, *T. koreensis*, *T. soliatarius*, and *T. osmophilus* [7]. Due to their halophilic nature, *Tetragenococcus* species get interests as starters for fermented foods with high salinities such as jeotgal and fish sauce [1, 8]. But studies on the genetics and genetic engineering of tetregenococci are still limited.

Plasmids from LAB such as *Lactobacillus*, *Leuconostoc*, and *Weissella* genera have been used as the frames for cloning vectors intended for the introduction and expression of genes in LAB [9-11]. However, few studies have been conducted on the plasmids from *Tetragenococcus* species, and few vectors have been constructed based on the plasmids from *Tetragenococcus* species. Construction of cloning vectors for *Tetragenococcus* species will be helpful not only for the basic studies on the genetics of *Tetragenococcus* species but also for the genetic engineering of *Tetragenococcus* species. A 8.7 kb cryptic plasmid (pUCL287) from *T. halophilus* ATCC33315 was characterized and used for vector construction. pUCL287 is replicating via theta-mode and its minimal replicon locates at a 1.2 kb fragment [12, 13]. *E. coli* - *Tetragenococcus* shuttle vectors were constructed based on the 1.2 kb minimal replicon of pUCL287 and pUC19 [14]. A cloning vector for LAB (pUBU) was constructed by using the 1.2 kb fragment as a replicon, and pUBU successfully replicated in several LAB genera with the transformation efficiencies of 10^3^ TFs/μg DNA [15].

In this study, a small cryptic plasmid, pTH32, was characterized from *T. halophilus* 32. *T. halophilus* 32 was isolated from jeotgal, Korean traditional fermented seafood. A new shuttle vector pMJ32E was constructed based on pTH32 and pBluescript II(+). pMJ32E successfully replicated in *Enterococcus faecalis* 29212 and *Tetragenococcus halophilus* 31. The result indicated that pMJ32E could be useful for the genetic engineering of *Tetragenococcus* and *Enterococcus* species. pMJ32E is a valuable addition to existing cloning vectors for LAB.

## Materials and Methods

### Bacterial Strains, Plasmids and Culture Conditions

Bacterial strains and plasmids used in this work are listed in Table 1. *Tetragenococcus* species were cultivated in lactobacilli MRS broth (Acumedia, USA) or agar (1.5%, w/v) containing NaCl (5%, w/v) under anaerobic conditions at 30°C. *Enterococcus*, *Lactobacillus*, *Lactococcus* and *Weissella* species were cultivated in the same media without NaCl. *Escherichia coli* DH5α was cultivated in Luria-Bertani (LB, Acumedia, USA) medium at 37°C with aeration. The following antibiotics were added to the media as selective agents when required: ampicillin (Sigma-Aldrich, USA) (100 μg/ml for *E. coli*) and erythromycin (Sigma-Aldrich, USA) (200 μg/ml for *E. coli*, 50 μg/ml for *Enterococcus* and *Tetragenococcus*, 5 μg/ml for *Lactobacillus*, *Lactococcus*, and *Weissella* strains).

### DNA Manipulations

Plasmid DNA was prepared from LAB by the method of O’Sullivan and Klaenhammer [19] and from *E. coli* DH5α using a commercial kit (FavorPrep plasmid extraction mini kit, Favorgen, Vienna, Austria). Restriction enzymes, T4 DNA ligase, and alkaline phosphatase (Promega, USA) were used according to the protocols provided by the suppliers. Polymerase chain reaction (PCR) amplifications were carried out with 10 to 100 ng DNA template and 10 pmol primers using *Ex Taq* DNA polymerase (Takara, Japan). Plasmid DNA and PCR amplicons were verified by agarose gels electrophoresis, stained with EcoDye DNA staining solution (Biofact, Korea) and visualized under UV light.

### DNA Sequence Analysis

pTH32 was digested with EcoRI and HindIII, and the resulting two fragments were individually cloned into pUC19. The nucleotide sequences of 2 fragments were determined by using M13 fwd (5′- GTTTTCCCAGTC ACGAC -3′) and M13 rev (5′- GCGGATAACAATTTCACACAG-3′) universal primers. The internal region of a larger fragment was sequenced by using pUCB-F (5′-ACAAGGCTTTGCAAGCCCAACGCA-3′) and pUCB-R (5′-ACAAACGGTTACGGGCTGCGTCAA-3′) primers. DNA sequencing was performed at Cosmogenetech (Korea). Analysis of the DNA sequence was performed primarily using the BLAST (https://blast.ncbi.nlm.nih.gov/Blast.cgi) and GenBank database. Open reading frame (ORF) prediction was performed using the ORF finder (http://www.ncbi.nlm.nih.gov/gorf/gorf.html). Promoter sequences were predicted using the ‘‘Neural Network Promoter Prediction’’ program of the University of Berkeley (http://www.fruitfly.org/seq_tools/promoter.html). Direct repeats and inverted repeats were predicted using Novoprolabs Repeats Finder for DNA/Protein Sequences (https://www.novoprolabs.com/tools/repeats-sequences-finder).

### Construction of pMJ32E and Transformation of LAB and *E. coli* DH5α with pMJ32E

pTH32 was digested with HindIII and then ligated with pBluescript II KS(+) (Stratagene, USA). The recombinant (pMJ32) was obtained. Erythromycin resistance gene (*ermC*) was PCR amplified from pJY33E by using primer pair: ErmC-F (5′- CCCTCTAGACTTATCGGATAATAA-3′, XbaI site underlined) and ErmC-R (5′-GGGGGATCCACAAAAAATAGGCAC-3′, BamHI site underlined) [11]. The amplified *ermC* was inserted into pMJ32 at XbaI and BamHI sites.

*E. coli* DH5α competent cells were prepared and transformed by standard protocols [20]. The plasmid DNA and *E. coli* competent cells were mixed, kept on ice for 1 min and transferred to a 0.1 cm electroporation cuvette. Electroporation was performed with a Gene Pulser II (BioRad, USA) under the following conditions: peak voltage, 2.1 kV; capacitance, 25 μF; resistance, 200 Ω. After pulsing, cells were resuspended in 1 ml of LB broth, incubated for 1 h at 37°C, and spreaded onto LB plates containing antibiotics. Competent cells of *Lactobacillus plantarum* KCTC3104, *Lactococcus lactis* subsp. *cremoris* MG1363, and *Weissella confusa* CB1 were prepared and transformed according to a previous report [21]. Competent cells of *Enterococcus* and *Tetragenococcus* genera were prepared and transformed according to a previous report [22].

### Expression of *pepA* from *T. halophilus* CY54

A *pepA* gene encoding aminopeptidase A (PepA) was PCR amplified from *T. halophilus* CY54 chromosomal DNA by using *pepA*-F (5′-CCCGTCGACTGCTGCTTCATAATG-3′, SalI site underlined) and *pepA*-R (5′-TTTCTCGAGCTGGCAACCTCAATC-3′, *XhoI* site underlined) primers [23]. The PCR amplified *pepA* was 2.6 kb in size, including its own promoter, and inserted into pMJ32E at SalI and XhoI sites. The resulting pMJ32E*pepA* was introduced into *T. halophilus* 31 and *E. coli* DH5α by electroporation, respectively. *T. halophilus* 31 and *E. coli* DH5α transformants (TFs) carrying pMJ32E*pepA* were grown in MRS broth (with 5% NaCl) and LB broth, respectively. Cells were harvested at the end of logarithmic growth phase by centrifugation (5,000 ×*g* for 15 min at 4°C), washed 3 times with phosphate-buffered saline (PBS) and resuspended in PBS. *T. halophilus* cells resuspended in PBS with lysozyme (10 mg/ml) were incubated for 1 h at 37°C before subjected to ultrasonication (Bandelin Electronic, Berlin, Germany). *E. coli* cells were disrupted by ultrasonication without lysozyme pretreatment. Disrupted cells were centrifuged at 12,000 ×*g* for 15 min at 4°C, and the supernatants were used as the cell extracts for PepA activity measurements. The protein concentration of the cell extract was determined by the Bradford method [24]. PepA activities in the cell extracts were measured according to previous report using glutamyl *p*-nitroanilide (glu-pNA) as the substrate [25]. One unit (U) of PepA was defined as the amout of enzyme that released 1 nmol of *p*-nitroaniline per minute under the assay conditions.

### Stability of pMJ32E in *T. halophilus* 31

Stability of pMJ32E was examined by a method as described previously [10]. *T. halophilus* 31 TFs harbouring pMJ32E were grown in MRS broth with NaCl (5%) and erythromycin (Em, 50 μg/ml) for 48 h at 30°C. Then the culture was used to inoculate (1%, v/v) fresh MRS broth containing NaCl (5%) but no Em, and the inoculated culture was incubated for 48 h at 30°C. Subculturing was repeated up to 7 times without Em selection. Aliquots of each culture were spreaded onto MRS plates containing NaCl (5%) only and MRS plates containing NaCl (5%) and Em (50 μg/ml), respectively. The percentage of cells harboring pMJ32E was calculated by dividing the number of cells on MRS plates with Em by the number of cells on MRS plates without Em, and multiplied by 100.

### Nucleotide Sequence

The complete nucleotide sequence of pTH32 was deposited to GenBank with the accession number MZ687077.

## Results and Discussion

### Characterization of pTH32

T. halophillus 32 was one of the isolates from jeotgal products, Korean traditional fermented seafoods by professor Lee group at Kyonggi university, Korea and kindly provided for this work. T. halophillus 32 possesses a small-sized plasmid, pTH32. Due to the small size, pTH32 was chosen as the frame for a *Tetragenococcus* – *E. coli* shuttle vector. pTH32 was digested with several restriction enzymes and it was found that pTH32 had a single recognition site for EcoRI, HindIII, NdeI, and NcoI. When pTH32 was double digested with EcoRI and HindIII, 2.2 and 1.0 kb fragments were generated. Each fragment was cloned into pUC19, resulting in pUCTH32L (pUC19 with 2.2 kb fragment) and pUCTH32S (pUC19 with 1.0 kb insert). The nucleotide sequences of the inserts were determined and combined into one sequence. pTH32 was 3,198 bp in length, and the G+C content was 35.84%, and it was within the known range of G+C contents of *Tetragenococcus* species [26].

**Fig. 1:**
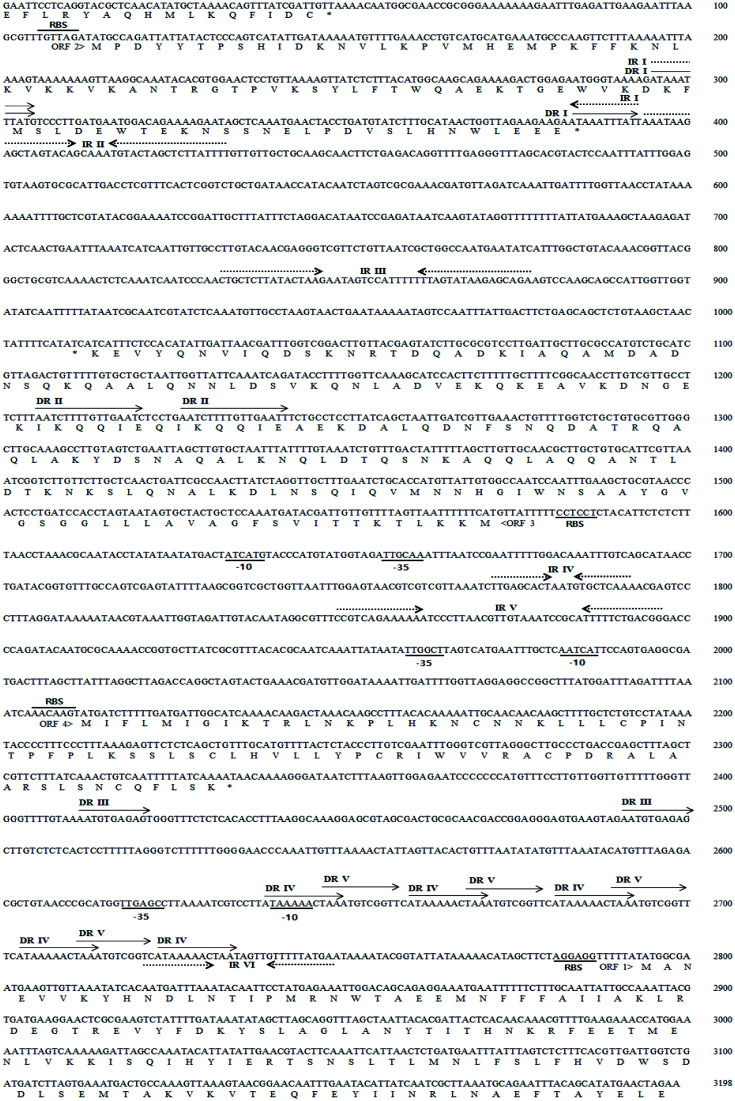
Nucleotide sequence of pTH32. The dotted arrow indicates inverted repeats (IRI-IRVI) shown above the corresponding sequences and the solid arrow indicates direct repeats (DRI-DRV) shown above the corresponding sequences. RBS (ribosome binding site) and putative promoter sequences (-35 and -10) are underlined. Amino acid sequence of ORFs are shown as a single letter under the corresponding nucleotide sequences.

Sequence analysis revealed the presence of 4 ORFs, 6 palindromic sequences (inverted repeat, IR I–VI), and 5 direct repeats (DR I–V) (Fig. 1). DRs and IRs observed in many plasmids are involved in the regulation of the replication processes of plasmids [27]. Many DRs and IRs are present upstream of *orf1*, the region where regulation of replication processes is expected to occur. *orf1* can potentially encode a protein of 151 amino acids showing 65.16% sequence similarity with a RepB family plasmid replication initiator protein from *Enterococcus faecium* (WP_159373655.1). *orf2* can potentially encode a protein of 90 amino acids showing 69.35% sequence similarity with a RepB family plasmid replication initiator protein from *Staphylococcus aureus* (WP_224670973.1). RepB proteins are known to have nicking-closing (topoisomerase I-like) activities for supercoiled DNA and are found among rolling circle replicating (RCR) plasmids [28]. *orf1* and *orf2* might form an operon since the distance between the end of ORF1 and the start of ORF2 is close, 62 nucleotides. Possible promoter sequences (-35 and -10) are located upstream of *orf1* (underlined in Fig. 1). IRII located immediate downstream of ORF2 might serve as a transcription terminator. It can form an hairpin-loop structure with the free energy of -15.86 kcal/mol as determined by mfold program (http://www.unafold.org/mfold/applications/dna-folding-form.php).

*orf3* can potentially encode a protein of 185 amino acids showing 98.38% sequence similarity with a hypothetical protein from *T. halophilus* (WP_012478268.1). *orf4* can potentially encode a protein of 74 amino acids showing 69.44% sequence similarity with a hypothetical protein from *T. halophilus* (WP_176446071.1). The functions of hypothetical proteins are unknown because they do not show any significant homologies to known proteins in the database. Considering the small size and significant homologies with RepB proteins, pTH32 was expected to replicate via RCR mode.

### Construction of pMJ32E and Transformation of LAB

**Fig. 2:**
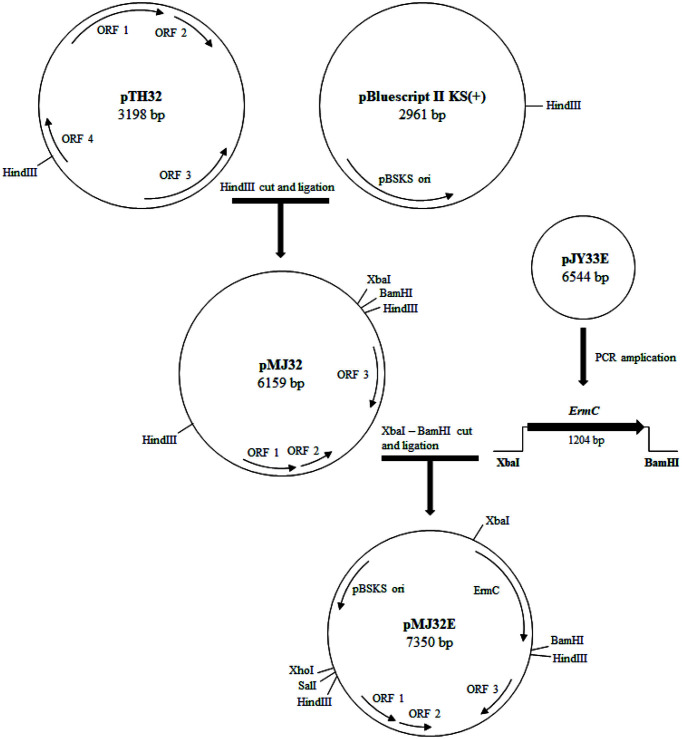
Schematic presentation of pMJ32E construction. pTH32 was digested with HindIII and ligated with pBluescript II KS(+), resulting in pMJ32. pMJ32 was double digested using XbaI and BamHI and ligated with a 1.2 kb *emrC* fragment from pJY33E, resulting in pMJ32E.

A *Tetragenococcus* - *E. coli* shuttle vector, pMJ32E, was constructed as described above (Fig. 2). Transformation of several LAB genera was tried with pMJ32E. pMJ32E successfully replicated in *Enterococcus faecalis* 29212 and *Tetragenoccus halophilus* 31. Transformation efficiency was 1.2 × 10^2^ and 1.1 × 10^1^ CFU/μg DNA for *E. faecalis* 29212 and *T. halophilus* 31, respectively. These efficiencies were lower than those reported for vectors based on pUCL287 replicon, which were in the range of 1.14 × 10^3^ – 2.1 × 10^4^ CFU/μg plasmid [14, 15]. *T. halophilus* 31 was isolated from jeotgal together with *T. halophilus* 32, and *T. halophilus* 31 harbors a 5 kb plasmid (results not shown). Among the several *Tetragenococcus* species tested, *T. halophiluds* 31 was the only strain transformed by pMJ32E. Transformation efficiency for *T. halophilus* 31 was quite low, but it could be improved if efforts to optimize the transformation conditions are tried. pMJ32E seems to have narrow host range since other LAB genera were not transformed with pMJ32E. Another possibility is that transformation of other LAB genera with pMJ32E might be more difficult compared with *Tetragenococcus* and other closely related LAB such as *Enterococcus*. More studies are necessary to clarify this issue.

### Expression of *pepA* from *T. halophilus* CY54 in *T. halophilus* 31

To test the potential of pMJ32E as a cloning vector for *Tetragenococcus* species, expression of *pepA* gene in a heterologous host was tried. pMJ32E*pepA* was constructed as described above (Fig. 2). *T. halophilus* 31 [pMJ32E*pepA*] showed PepA activity of 49.8 U/mg protein, which was much higher than 9.1 U/mg protein of *T. halophilus* 31 harboring intact pMJ32E (control). *E. faecalis* 29212 [pMJ32E*pepA*] showed PepA activity of 39.2 U/mg protein, which was much higher than 20.2 U/mg protein of *E. faecalis* 29212 harboring intact pMJ32E (control). *E. coli* DH5α [pMJ32E*pepA*] showed PepA activity of 380 U/mg protein, which was also higher than 94 U/mg protein of *E. coli* DH5α harboring pMJ32E (control) (Table 2). The results proved the usefulness of pMJ32E as a cloning vector for *T. halophilus* and *E. faecalis*.

### Stability of pMJ32E in *T. halophilus* 31

**Fig. 3:**
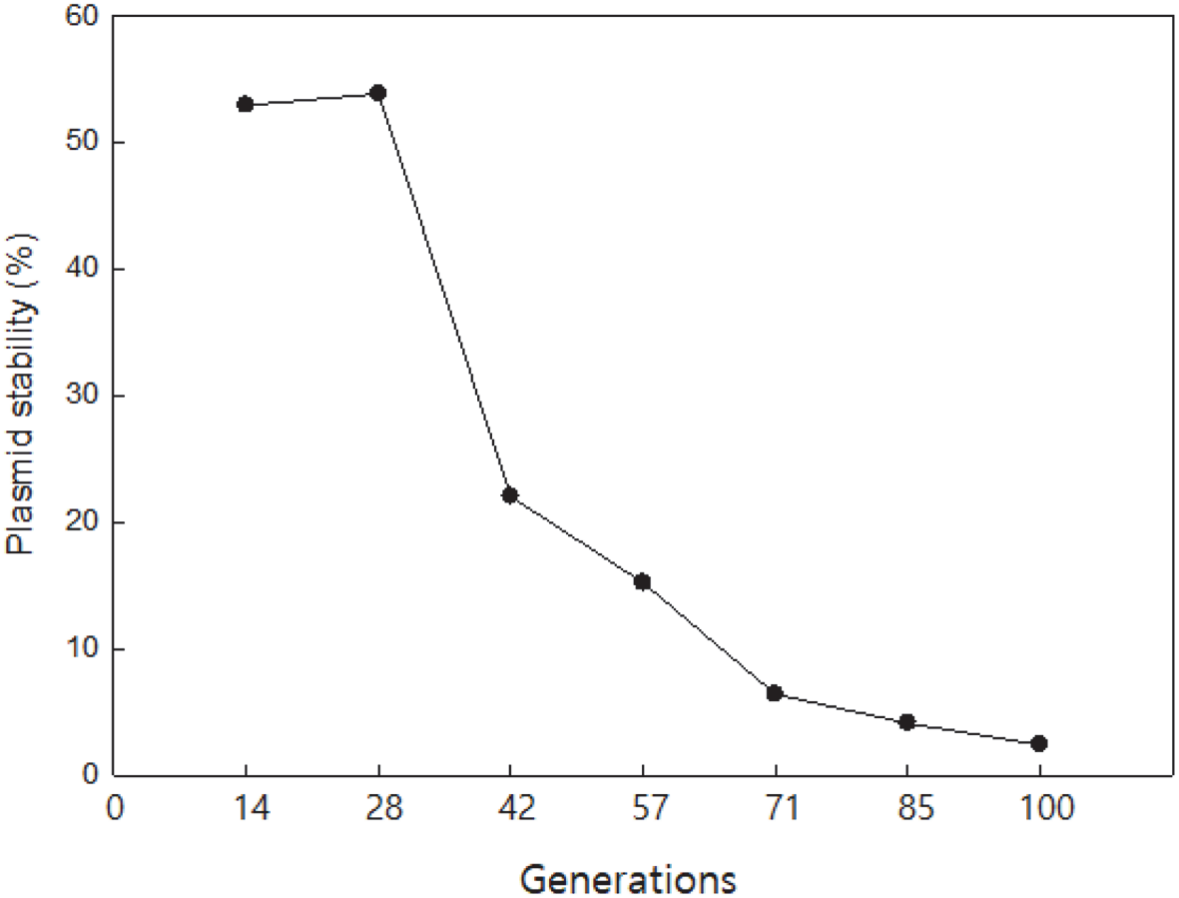
Stability of pMJ32E in *T. halophilus* 31 during extended growth in MRS broth with 5% NaCl but without Em.

The segregational stability of pMJ32E in *T. halophilus* 31 was monitored under the absence of Em selection (Fig. 3). After the first 28 generations, 53.8% cells retained pMJ32E and the portion of cells which lost pMJ32E increased rapidly thereafter. After 100 generations, only 2.4% of the cells still retained pMJ32E. The results are comparable to those observed among many RCR-type plasmids. RCR plasmids usually have low segregational stabilities due to the accumulation of ssDNA intermediates [27]. Theta type plasmids, which do not produce ssDNA intermediates, are more stable than RCR plasmids [27]. For example, a shuttle vector pFBYC018E based on a RCR plasmid (pFR18) showed just 3% stability after 100 generations [29]. pL11 based on another RCR plasmid (pSMA23) showed 2% and 6% stability in *Lb. casei* and *Lb. gasseri*, respectively, after 210 generations [30]. Whereas pSJ33E, a theta-replicating plasmid from *Leuconostoc mesenteroides* SY2, was maintained 100% in *L. mesenteroides* SY1 after 100 generations [10]. A shuttle vector, pRCEID-LC7.6, constructed by ligation of a theta replicating plasmid from *Lactobacillus casei* with pUC19 showed 16% stability in *L. casei* RCEID02 after 200 generations [31]. The segregational stability of pMJ32E in *T. halophilus* 31 was lower than those of theta-replicating vectors but similar to those of RCR plasmids, supporting the rolling circle replication of pMJ32E.

**Fig. 4:**
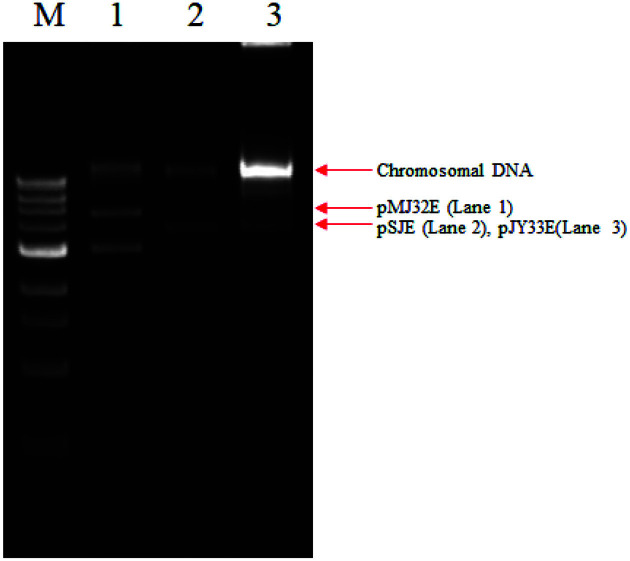
Comparison of the copy number of pMJ32E with other vectors. M, iVDye 1kb DNA ladder (GenDEPOT, USA); 1, pMJ32E isolated from *T. halophilus* 31; 2, pSJE isolated from *Levilavtobacillus brevis* 2.14; 3, pJY33E isolated from *Levilavtobacillus brevis* 2.14.

The copy number of pMJ32E in *T. halophilus* 31 was qualitatively compared with those of pSJE (a theta-type plasmid) in *Levilavtobacillus brevis* 2.14 [10] and pJY33E (a RCR-plasmid) in *L. brevis* 2.14 [11]. Three cultures were grown in MRS broth (10 ml) under the same conditions. When the OD_600_ reached 0.6, plasmid DNA was prepared and dissolved in 40 μl distilled water. The same amount of DNA samples (5 μl) were analyzed by agarose gel electrophoresis and stained with ethidium bromide (Fig. 4). The results indicated that the copy number of pMJ32E was similar with those of pSJE and pJY33E.

A small cryptic plasmid, pTH32, was isolated from *T. halophilus* 32 and characterized. A novel *E. coli* –*Tetragenococcus* shuttle vector, pMJ33E, was constructed based on pTH32. pMJ32E successfully replicated in *T. halophilus* 31 and *E. faecalis* 29212. But no TFs were obtained for other LAB genera, which might be due to the narrow host range of pTH32 or failure to find optimum transformation conditions. A *pepA* gene from *T. halophilus* CY54 was successfully expressed in a heterologous host, *T. halophilus* 31 and *En. faecalis* 29212 by using pMJ32E, and high level of PepA activity was observed in a TF. The result showed that pMJ32E could be useful for genetic engineering of *Tetragenococcus* and *Enterococcus* species.

## Figures and Tables

**Table 1:** Bacterial strains and plasmids used in this study.

Bacteria and plasmids	Description	Reference
*Escherichia coli* DH5α	F− φ80lacZΔM15 Δ(*lac*ZYA-*arg*F)U169 recA1 *end*A1 *hsd*R17(r_k_^−^, m_k_^+^) *pho*A *sup*E44 *thi*-1 *gyr*A96 *rel*A1 λ^−^	Gibco BRL
*Tetragenococcus halophilus* 31	Transformation host	Kyonggi university
*Tetragenococcus halophilus* 32	Source of pTH32 native plasmid	Kyonggi university
*Enterococcus faecalis* 29212	Lab strain, transformation host	This study
*Lactobacillus brevis* 2.14	Sak^−^ Imm^−^, indicator strain for Sakacin A	[16]
*Lactobacillus plantarum* KCTC3104	Transformation host	[17]
*Lactococcus lactis* subsp. *cremoris* MG1363	Lac^−^,Gal^−^, plasmid-free and prophage-cured derivative of NCDO712	[18]
*Weissella confusa* CB1	Lab strain, transformation host	[17]
Plasmid		
pBluescript II KS (+)	*E. coli* cloning vector, 2.96 kb, Apr , *lac*Z	Stratagene
pTH32	cryptic plasmid from *T. halophilus* 32, 3.2 kb	This study
pSJE	*E. coli* – *Leuconostoc* shuttle vector, 6.6 kb, Em^r^	[10]
pJY33E	*E. coli* – *Weissella* shuttle vector, 6.5 kb, Ap^r^, Em^r^	[11]
pUCTH32L	pUC19:: 2.2 kb EcoRI-HindIII fragment from pTH32, 4.9 kb, Ap^r^	This study
pUCTH32S	pUC19:: 1 kb EcoRI-HindIII fragment from pTH32, 3.7 kb, Ap^r^	This study
pMJ32	pBluescript II KS (+):: 3.2 kb HindIII digested pTH32, 6.2 kb, Ap^r^	This study
pMJ32E	pMJ32:: 1.2 kb *emrC* from pJY33E, 7.3 kb, Ap^r^, Em^r^	This study
pMJ32EpepA	pMJ32E:: 2.6 kb *pepA* from *T. halophilus* CY54 chromosomal DNA, 10 kb, Ap^r^, Em^r^	This study

**Table 2:** Aminopeptidase A activities of *E. coli*, *T. halophilus* and *E. faecalis* TFs.

Strains	Total activity (U/mg protein)
*E. coli* DH5α [pMJ32E]	94
*E. coli* DH5α [pMJ32EpepA]	380
*T. halophilus* 31 [pMJ32E]	9.1
*T. halophilus* 31 [pMJ32EpepA]	49.8
*E. faecalis* 29212 [pMJ32E]	20.2
*E. faecalis* 29212 [pMJ32EpepA]	39.2
